# Using Open Public Meetings and Elections to Promote Inward Transparency and Accountability: Lessons From Zambia

**DOI:** 10.34172/ijhpm.2020.84

**Published:** 2020-06-23

**Authors:** Taryn Vian, Rachel M. Fong, Jeanette L. Kaiser, Misheck Bwalya, Viviane I.R. Sakanga, Thandiwe Ngoma, Nancy A. Scott

**Affiliations:** ^1^School of Nursing and Health Professions, University of San Francisco, San Francisco, CA, USA.; ^2^Department of Global Health, Boston University School of Public Health, Boston, MA, USA.; ^3^Department of Research, Right to Care Zambia, Lusaka, Zambia.; ^4^Department of Programs, Amref Health Africa, Lusaka, Zambia.

**Keywords:** Governance, Transparency, Democratic, Community-Based, Maternity Waiting Homes

## Abstract

**Background:** Community-led governance can ensure that leaders are accountable to the populations they serve and strengthen health systems for maternal care. A key aspect of democratic accountability is electing respective governance bodies, in this case community boards, and holding public meetings to inform community members about actions taken on their behalf. After helping build and open 10 maternity waiting homes (MWHs) in rural Zambia as part of a randomized controlled trial, we assisted community governance committees to plan and execute annual meetings to present performance results and, where needed, to elect new board members.

**Methods:** We applied a principally qualitative design using observation and analysis of written documentation of public meetings to answer our research question: how do governance committees enact inward transparency and demonstrate accountability to their communities. The analysis measured participation and stakeholder representation at public meetings, the types and purposes of accountability sought by community members as evidenced by questions asked of the governance committee, and responsiveness of the governance committee to issues raised at public meetings.

**Results:** Public meetings were attended by 6 out of 7 possible stakeholder groups, and reports were generally transparent. Stakeholders asked probing questions focused mainly on financial performance. Governance committee members were responsive to questions raised by participants, with 59% of answers rated as fully or mostly responsive (showing understanding of and answering the question). Six of the 10 sites held elections to re-elect or replace governance committee members. Only 2 sites reached the target set by local stakeholder committees of 50% female membership, down from 3 at formation. To further improve transparency and accountability, community governance committees need to engage in advance preparation of reports, and should consult with stakeholders on broader measures for performance assessment. Despite receiving training, community-level governance committees lacked understanding of the strategic purpose of open public meetings and elections, and how these relate to democratic accountability. They were therefore not motivated to engage in tactics to manage stakeholders effectively.

**Conclusion:** While open meetings and elections have potential to enhance good governance at the community level, continuous training and mentoring are needed to build capacity and enhance sustainability.

## Background

**Key Messages**

**Implications for policy makers**

- Strengthening health systems to foster community-level governance can lead to improved maternal, neonatal and child health, and open public meetings and elections are an important aspect of shared control and accountability.
- Meeting participation, stakeholder representation, community engagement, and responsiveness of the governance committees are key indicators of transparency and accountability through open meetings and elections.
- Capacity building for community-level governance and accountability is a long-term process. Governments and development partners should anticipate the need for ongoing training and mentorship to implement such structures for maternity waiting homes (MWHs). This is necessary to improve the likelihood that community governance will be sustained.

** Implications for the public**

Local people who serve on governance committees for health programs such as maternity waiting homes (MWHs), need to explain to the rest of the community how they make decisions and use money. This can happen during open community meetings where anyone can share opinions, ask questions, and get answers. At these open meetings, people should vote to re-elect committee members who are doing a good job or replace committee members who are not. This is democratic accountability, and it can be a way to assure that the people meant to represent community interests really are doing that. It can take a long time for this process to work smoothly, and will require a lot of effort and possibly some outside assistance. But over time, governance committees can become more accountable to the people, and health outcomes can improve.

Strengthening health systems to foster community engagement and empowerment can lead to improved maternal, neonatal and child health.^1-3^ An important aspect of strengthening community participation is assuring good governance, systems of “shared control” and accountability.^1,4^ Community-led governance helps ensure that leaders are answerable and accountable to the populations they serve.^5,6^ Those in a position of authority—community leaders—are obligated to provide information about or justification for their actions to other community members, and should face consequences if they fail to respond to the needs and concerns of their constituencies.^7^ Community leaders can include traditional authorities, religious figures, representatives of secular government at the local level, and members of established community groups, such as local school councils or health committees. Although many studies have documented mechanisms to improve accountability for maternal and newborn care at the national or health facility level, few have focused specifically on mechanisms to hold community-level committee members accountable.^8,9^

Two key aspects of accountability are holding elections, and holding open public meetings so that policies are informed and overseen/questioned by broad discussion and agreement.^10^ In many settings, democratically-elected community members are more likely to gain public trust and acceptance of decisions.^10-12^ Through public meetings, leaders have an opportunity to interact with people they represent, and citizens can convey opinions and influence agendas.^13^

The implementation challenges of community-led governance are not well understood. Studies have focused on the internal operations of governance boards,^14^ and how the boards may influence organizational strategies and outcomes,^15-17^ but few studies examine election processes or “inward transparency” facilitated through community governance structures,^18^ that is, enabling the community to see into the organization through open meetings and interactions with board members. An analysis of nonprofit boards, which often involve community members, may provide insights to lack of inward transparency. For example, a study of 300 non-governmental organizations (NGOs) in Uganda found that 90% of NGOs had a board overseeing activities, and 80% claimed that they prepared an annual report and financial statement; however, many NGOs were not up-to-date in their reporting.^19^ Moreover, while virtually all the NGOs claimed to hold public meetings with members or beneficiaries, researchers suspected that only a few NGOs actually circulated figures and reports that were accurate, detailed and current.^19^ In Bangladesh, researchers found that lack of pragmatic planning, weak training of governance committee members, and poor communication with the larger community hindered community participation at meetings.^6^ In addition, issues of power asymmetry lessen the likelihood that female committee members, or low status members of the community at large, will speak up.^4^ Community governance structures need to facilitate sharing of information across hierarchies, and to assure context-specific representation of different interests.^8,20^

This article adds to our understanding of inward transparency and its relationship to accountability through a case study of community governance of maternity waiting homes (MWHs) in Zambia. MWHs are residential facilities located next to a health facility where a pregnant woman can stay during the final weeks of her pregnancy. The Maternity Homes Access in Zambia Project was a randomized controlled trial designed to measure the impact of a quality MWH model on facility delivery among women living farthest (≥10 km) from their designated health facility in rural Zambia.^21^ In addition to improving the MWH infrastructure and providing linkages to primary health services, including skilled delivery care, the project helped to increase community participation and ownership. With extensive local consultation, a new locally-led governance structure was created to strengthen capacity and increase accountability. Consultation was required to better understand and integrate the new governance responsibilities with any existing structures for decision-making in the communities, such as neighborhood health committees and the traditional chiefdom leadership structures (as described in the Methods section).

 A governance committee with an average of 9.3 members was established at each of the 10 study sites. Each governance committee included an executive committee (chair, vice chair, treasurer, and secretary). The governance committee was responsible for setting policies and strategies to promote the MWH mission and for overseeing policy implementation in the interest of the beneficiaries and the community at large. The governance committee was also responsible for mobilizing and using community resources for the sustainability of the MWH. This included oversight to safeguard MWH assets and ensuring that financial reports accurately presented its financial condition.

 After the MWHs had operated for about 24 months, project staff assisted the governance committees to plan and execute an annual general meeting (AGM) to present financial and performance results to the larger community and, where needed, to elect new board members. This study examines the experience of holding the first AGMs and elections in the 10 sites between February and March 2018. Our objective is to explore whether the governance committees practiced inward transparency and were held accountable through elections and open public meetings, and to examine the barriers and facilitators to open meetings and election processes as a strategy for good governance of the local committees.

###  Theoretical Frameworks

We used 2 theoretical frameworks to guide our study. First, we applied Andrews and Shaw’s framework of good local governance to guide the establishment of the governance committees. The elements of Andrews and Shaw’s framework include: (1) conform to legislation; (2) maintain fiscal health; (3) do the right things (responsive); (4) do them in the right way (efficient); and (5) be accountable to constituents for process, outputs, and outcomes.^21^

In addition, we used Derick Brinkerhoff’s health governance framework (Table 1), to code and analyze questions raised at the AGMs, by type and purpose of accountability.^7^ Brinkerhoff includes 3 types of accountability: financial, performance, and democratic/political accountability. All 3 types of accountability refer to adherence to policies, procedures, norms and standards for proper use of authority. Financial accountability focuses on controlling and reporting use of financial resources and making progress toward sustainability; performance accountability considers documented results compared to plans, and institutional learning to support effective services; and democratic/political accountability focuses on representation, legitimacy, and responsiveness to community needs. An important part of democratic/political accountability is to engage stakeholders.^8^ It is important for community members to engage in discussion and ask questions at the AGM. In this way, they can assess whether governance committee members are responding to their expressed concerns.

**Table 1:** Theoretical Accountability Framework Guiding the Analysis of the Annual General Meetings

**Accountability Type**	**Purpose**
Financial: proper recording, disbursement, and use of financial resources	Adhere to policies and procedures for record keeping and financial controls; prepare proper and timely reports Control costs; control waste and corruption Ensure progress toward financial stability
Performance: support management and effective service delivery	Adhere to policies, procedures, norms and values Document performance Compare results to targets and report reasons for variance Organizational learning; question if goals should change
Democratic/political: ensure responsible officials deliver on promises and respond to the needs of constituents	Ensure community satisfaction with results achieved Ensure representativeness and legitimacy of governance committee members vis à vis constituents (elections) Adhere to procedures, norms, values and proper use of authority

Framework adapted from Brinkerhoff’s analytic framework for mapping accountability.^7^

## Methods

###  Study Setting

Community governance structures were established in 10 randomly selected primary health facility study sites in the rural districts of Choma, Kalomo, and Pemba of Southern province; and Nyimba district of Eastern province. Further information on the study site eligibility criteria and random selection process can be found elsewhere.^21^ The rural health facility catchment area population sizes range from 5000 to 11 000 with an average of 49 villages, each an average 9.7 km from the health facility.

In setting up the governance structures, the project worked with local stakeholders, including traditional and secular government leaders. The goal was to ensure local ownership of design decisions that would set initial guidelines for the structures. Zambia has a traditional leadership structure based on chiefdoms with their own set of geographical boundaries that exists outside of the secular government structures. The chief is considered the custodian of the natural resources, facilitator of social development for their people, responsible for settling disputes among their people, and guardian of traditional norms, culture, beliefs and values. Individuals living within a chiefdom are subjects of the chief, which is a hereditary position. The traditional leader within each village, the village headman, is accountable to the chief and responsible for carrying out the chief’s duties at the village level. There are multiple levels of leaders between chiefs and village headmen, including senior headmen, section chairmen, and chief’s representatives. Traditional leaders in Zambia have routinely been engaged in MCH programs and are seen as local champions. The policies they put in place can impact community actions: for example, in some localities, traditional leaders require women to make a payment to the chief if they have a home delivery (this is meant to encourage facility-based deliveries).^22^

 Separate from the traditional leadership structure is the secular government structures, the lowest level of which is the ward, led by an elected civic councilor. Wards and their associated secular government structures are aggregated into constituencies, districts and provinces, and then at the national level.

###  Background on the Intervention

####  Governance Committee Structures and Initial Capacity Building

To assure that we met Andrews and Shah’s first criteria for good governance (conform to legislation), we made efforts to adhere to the formal rules for managing a collaborative in Zambia, as well as respecting the informal customs of starting a community-owned facility within a chiefdom. We investigated local regulations to ensure that committees had legal status and were properly registered. Consulting widely to determine who was influential in the community,^23^ project staff created short-term, district-level steering committees composed of traditional chiefs, health facility staff, ministry officials, local community leaders, and reproductive-aged women, and identified some decisions the committees should consider, such as establishing terms of reference for the governance committees and processes for member selection. Aligned with stakeholder steering committee recommendations, project staff then facilitated the formation of the governance committees at each study site. The stakeholder steering committees advised that governance committees should have representation from villages in the health facility catchment area, community health workers, health facility staff, and traditional leadership; they emphasized a minimum of 50% female representation, in an effort to assure that structures meant to improve women’s health could have leadership representative of the target population. Each site was given the flexibility to adapt recommended guidelines and establish their own criteria for committee membership. For example, one committee decided to hold quarterly stakeholder meetings instead of only holding one annual meeting. Project staff monitored the process. Governance committees were created between January and May 2016, after which the initial steering committees were dissolved. Project staff helped each governance committee to choose a management unit, usually an individual or several individuals in charge of day-to-day MWH management.

To meet Andrews and Shah’s other criteria (fiscal health, responsiveness, efficiency, and accountability), project staff provided training to governance committee and management unit members in management and financial skills. Committee and management unit members also attended 2 lesson-learned workshops and project staff conducted routine mentorship visits over 24 months. Additionally, project staff supported sites to develop MWH operating systems, including a financial system, described in further detail elsewhere.^24^ To further reinforce fiscal health and sustainability, project staff helped the sites to choose income-generating activities (IGAs). The project provided resources and training for a tailoring business to all sites, and one additional business of their choosing (raising goats, running an agricultural supply store, or running a grinding mill). After paying expenses of the IGA, the profit from the IGA was put in a bank account by the committee treasurer. These funds were available to pay operating expenses of the MWH. The governance committee provided oversight through 3 subcommittees: MWH operations, Finance, and IGA oversight. The subcommittees included 2-3 committee members, and met periodically to discuss specific issues related to their content areas.

####  Annual General Meetings and Elections

 To reinforce democratic accountability, we created ways for the committees to gather community input. We asked each site to hold an AGM after at least one full year of operation, and a re-election of committee members. The open meeting approach promotes transparency and allows communities to hold the governance committee accountable for having used their power and resources wisely. In advance, the governance committees in each site held meetings to identify stakeholders to invite and set the meeting agenda. Each site was asked to create an activity report to document the MWH’s activities and results, and a financial report to document financial performance of the MWH and IGAs; these reports were to be discussed at the preparatory meeting and presented to the community at the AGM.

###  Study Design

 We applied a mainly qualitative study design using observation and analysis of written documentation. Two members of our project team observed the meetings held in 9 MWH sites (due to timing, the team was unable to attend the meeting in one site). The project team members took handwritten notes that were later transcribed. We also reviewed the minutes and attendance register of the meeting where observers were not present, and reviewed reports of follow-up meetings organized at selected sites. We refer to sites by letter (A, B, etc) to maintain confidentiality.

 To assess democratic accountability, we analyzed meeting participation and stakeholder representation, transparency of the reporting and election processes, stakeholder engagement, responsiveness of committees, and change in committee composition.

###  Meeting Participation and External Stakeholder Representation

We calculated median attendance from sign-in sheets. We identified 7 possible external stakeholder groups (traditional leadership, health facility staff, health volunteers, church representatives, school staff, government officials, and other regular community members; described in Supplementary file 1) who had been invited to attend the AGM. We calculated the percent represented at the meeting by at least 1 attendee per group. If an individual represented 2 groups, the chairperson asked that he or she only state one group. Calculations of participation proportions, medians, and ranges for stakeholder groups in attendance were conducted in Microsoft^®^ Excel.

 We excluded the 86 members of the governance committees and MWH management units. While they are also stakeholders, we were primarily interested in examining the inward direction of transparency (when those outside an organization can see what is going on within). In this sense, we considered the governance committee members and MWH management unit staff to be insiders, rather than external stakeholders.

####  Transparency of Reporting and the Election Processes

We assessed the transparency of reporting through review of 3 documents that were intended to be presented at each meeting: the AGM Agenda, Financial Report, and Activity Report. We measured conformance to the standards enumerated in training and discussed during preparatory meetings (see Supplementary file 2). The agenda and activity reports each had 10 standards (for example, the agenda needed to include time for opening remarks, presentation and discussion of each specific report, while the activity report needed to include the reporting period, planned activities, implemented activities, challenges). The financial report had 5 standards (for example, the report should include a list of types and amounts of income, profit and loss statement). Each standard was rated as ‘yes’ (present) or ‘no’ (absent). Mean transparency scores for each domain were calculated in Microsoft^®^ Excel.

 For election transparency, we observed whether the site had articulated to the stakeholders the criteria for nominating and selecting committee members, and how the election process would work. We also observed whether an election was held. If it was not, we asked committee members for the reason.

####  Stakeholder Engagement 

 To measure stakeholder engagement, we reviewed the written meeting notes and extracted 67 questions asked by external stakeholders at the 9 meetings. We coded these questions according to the type of accountability (financial, performance, democratic) and the purpose of accountability, using our theoretical framework. Two researchers independently coded all questions, then met to discuss and reconcile areas of disagreement. We added one additional accountability type, “Information,” to code simple questions asked by participants about the basic structure of the MWH and its associated IGA. Examples of informational questions include “What does ‘IGA’ stand for?” and “Who are the members of the governance committee?” When questions fell into more than one category, we coded them to both categories.

####  Responsiveness of Governance Committee

We analyzed how the governance committee members answered questions posed by external stakeholders by looking for 3 criteria: demonstrated understanding of the question, a straightforward and clear answer, and justification or additional detail to support the answer. One researcher coded each answer according to these 3 criteria, rating individual criteria as not responsive, somewhat responsive, mostly responsive, or fully responsive. The researcher then assigned an overall responsiveness rating (Table 2). For the response to be considered fully responsive, the respondent must have shown an understanding of the question, provided a straightforward, clear answer, and provided justification or detail in relation to the question. A second researcher reviewed the coding and the 2 researchers discussed and resolved areas of disagreement. We excluded 5 questions that sounded more like suggestions.

**Table 2:** Responsiveness Rating Used to Systematically Assess Governance Committee Responses to Stakeholder Questions at the Annual General Meetings

**Responsiveness Rating**	**Criteria**
Not responsive	Did not show understanding of question, did not provide straight forward or clear answer, did not provide justification or detail.
Somewhat	Showed understanding of question, and partly answered the question. Answer may have been unclear or lacked justification or detail.
Mostly	Showed understanding of question, and answered the question. The answer may have lacked clarity or some justification.
Fully	Shows understanding of question, provided straight forward, clear answer, and provided justification or detail in relation to the question.

####  Governance Committee Composition

Project staff collected a register of governance committee membership which included basic demographics at the time of committee formation and approximately 24 months later, just after the AGMs and re-elections. The governance committee terms of reference state that a member shall be elected to a term of 12 months of service. However, the first AGMs were held at 24 months because the MWHs did not open until 6 months after committee formation and the IGAs were not operational until 12 months after committee formation. Proportions were calculated for respondent gender, health facility representation, traditional leadership representation, and members’ occupation. Means and standard deviations were calculated for committee members, highest grade completed, and overall female representation. All calculations were conducted in Microsoft^®^ Excel. Approximately 12% of all committee members over the 2 time points were missing data on the highest grade they completed in school.

 Some project staff provided technical assistance, as well as engaging in research. This may have introduced observer bias, when the researcher subconsciously projects his/her expectations onto the research. To limit this bias, the 2 researchers who analyzed data on transparency, stakeholder engagement, and responsiveness were not involved in on-the-ground activities.

## Results

###  Initial Committee Selection and Composition

Table 3 reflects the composition and characteristics of the governance committees upon their formation and after the AGMs approximately 24 months later. The communities initially selected committee members in one of 3 ways: (1) Community elections wherein community members selected from a list of candidates nominated by the stakeholder steering committee (n = 4 ); (2) Village headmen (traditional leaders) nominations, the headmen identified candidates based on qualification criteria from the stakeholder steering committee (n = 3); or (3) Health facility staff identified and selected members based on their geographic representativeness, availability, and prior involvement in health facility-related activities (n = 3).

**Table 3:** Characteristics of Governance Committees at Formation and 24 Months Later, by Site and Overall (n = 10)

	**Overall Mean (SD)**	**Site A**	**Site B**	**Site C**	**Site D**	**Site E**	**Site F**	**Site G**	**Site H**	**Site I**	**Site J**
Committee member selection mode	n/a	Health facility appointed	Headmen appointed	Community elected	Community elected	Headmen appointed	Health facility appointed	Community elected	Community elected	Health facility appointed	Headmen appointed
**At Formation**											
Number of GC members	9.3 (1.7)	9	10	7	6	9	10	12	10	10	10
Female representation, No. (%)^a^	39.3 (10.9)	3 (33.3)	3 (30.0)	2 (28.6)	2 (33.3)	4 (44.4)	5 (50.0)	4 (33.3)	3 (30.0)	5 (50.0)	6 (60.0)
Traditional leadership representation, No. (%)^a^	8.6 (6.9)	0 (0.0)	1 (10.0)	0 (0.0)	1 (16.6)	1 (11.1)	0 (0.0)	1 (8.3)	1 (10.0)	1 (10.0)	2 (20.0)
Highest grade completed, mean (SD)	9.6 (2.4)^b^	11.4 (2.2)^c^	9.7 (2.5)	9.1 (2.0)	10.3 (1.9)^d^	9.4 (2.8)	11.0 (2.0)	10.3 (2.0)	9.3 (1.8)	8.6 (2.3)	8.7 (2.7)
**24 Months After Formation**											
Re-election results	n/a	No re-election	Elected all new members	No re-election	Elected some new members	Re-elected all previous members	No re-election	Elected some new members	No re-election	Elected some new members	Elected some new members
Number of GC members	9.3 (1.6)	6	10	10	9	8	10	12	9	10	9
Female representation, No. (%)^a^	37.7 (8.4)	2 (33.3)	4 (40.0)	3 (30.0)	3 (33.3)	3 (37.5)	5 (50.0)	3 (25.0)	4 (44.4)	5 (50.0)	3 (33.3)
Traditional leadership representation, No. (%)^a^	6.33 (8.9)	0 (0.0)	1 (10.0)	2 (20.0)	0 (0.0)	0 (0.0)	0 (0.0)	0 (0.0)	1 (11.1)	0 (0.0)	2 (22.2)
Highest grade completed, mean (SD)	9.9 (2.4)^e^	10.5 (2.5)	10.3 (2.8)	9.5 (1.9)	11.4 (1.1)	9.8 (2.9)	11.0 (1.7)	10.4 (1.8)^f^	9.7 (2.1)	9.4 (2.3)	7.8 (3.8)^g^

Abbreviations: SD, standard deviation; GC, Governance Committee. ^a^ Mean proportion for all 10 sites for female representation and traditional leadership (n = 10); ^b^Missing 6.6% of data (n = 6); ^c^Missing 44.4% of data (n = 4); ^d^Missing 33.3% of data (n = 2); ^e^Missing 5.3% of data (n=5); ^f^Missing 16.7% of data (n=2); ^g^Missing 33.3% of data (n = 3).

 Upon initial formation, the governance committees had an average of 9.3 members. The average proportion of female members per committee was 39%, with 3 of the 10 sites having at least 50% female representation on the committee. No sites using the community election method to select the governance committees achieved the 50% minimum target, while the majority of sites that used health facility appointments achieved the 50% target. Seven of the 10 sites had representation from traditional leadership, and 100% of sites had at least one health facility representative. Fifty percent of sites also had a clergy member on the committee, while the majority of the remaining members were subsistence farmers (data not shown).

###  Annual General Meeting Participation and Representation 

 On average, 36.2 external stakeholders attended the AGM at each site. Mean attendance per site included about 15.2 representatives of traditional leadership (headmen, senior headmen, chief representatives, and section chairmen); 11.3 health volunteers (including members of Neighborhood Health Committee, Safe Motherhood Action Group, and community health workers); 2.5 church representatives; 1.4 teachers; 0.5 local government representatives; and 3.4 other local community members (eg, farmers, people without a listed occupation; Figure). The median number of stakeholder groups represented per site was 6 out of the possible 7 groups (range: 4-7).

**Figure:**
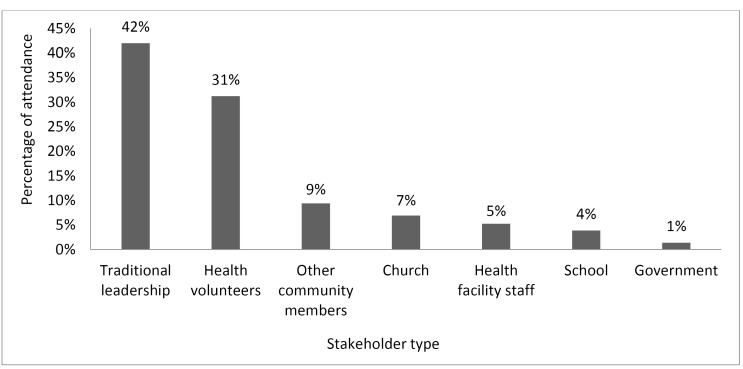
External Stakeholder Participation at Annual General Meetings.

Notes: See Supplementary file 1 for further explanation of stakeholders’ role in community health and development. Traditional Leadership grouping includes chief representatives, senior headmen, headmen, and section chairmen; Health Volunteers includes members of the Neighborhood Health Committee, Safe Motherhood Action Group, and community health workers; Government includes civic counselor at ward level; Other community members includes farmers and those who did not state occupation or affiliation.

###  Report Transparency

Table 4 shows the transparency of reporting against the standards.

**Table 4:** Transparency of 10 Maternity Waiting Home Reports Presented at Annual General Meetings

	**Actual Average Points**	**Total Possible Points**	**Average Score ( Average Points/Possible)**	**Actual Range**
Meeting Agenda	7.6	10	76%	5-10
Activity (Chairperson's) report	6.3	10	63%	0-10
Financial (Treasurer's) report	4	5	80%	1-5

Note: See Supplementary file 2 for the criteria. Each criterion was worth 1 point.

- *Agenda*. The mean score for agenda transparency was 7.6 out of 10. Meeting preliminaries such as self-introductions and opening remarks were a common feature in all sites. All sites also included time to present and discuss the financial report, and 9 of 10 sites included time to present and discuss the activity report.
- *Activity report*. The mean transparency score for the activity report was 6.3 out of 10. Eight sites had a written report available at the meeting, one site (J) provided a verbal report only, and one site (F) did not provide either written or verbal. All committee members were literate and sites were capable of written reporting. Most sites described implemented activities, but only one site (C) compared actual to planned activities and explained why some activities were not implemented.
- *Financial report.* The median score was 4 out of 5. All sites included the reporting period, and all but one included sources of revenue and expenditure. Only 3 sites calculated profit or loss.

Two sites gave verbal financial and activity reports, and left out many components in their verbal presentations. Written reports were generally more complete. All sites except one voted at the AGM to accept the activity report. Eight of 10 sites voted to accept the financial report. In the sites that did not accept reports, they asked the chairperson or treasurer to revise the report to include more detail, and held an extra meeting to vote on the report.

###  Election Transparency

 The election process was transparent in 6 out of 10 sites. These were characterized by clear explanations given to stakeholders for how the election process would work, and clear criteria presented to community members on how they should think about nominating and voting for governance committee members. Two sites (D, G) used a community selection process (wherein community members select from a list of people nominated during the meeting), and 2 sites used a 2-staged selection process involving headmen, who first gave their recommendations before the community selection process took place (B, E). Finally, 2 sites used a 2-staged process where health facility staff first selected an agreed upon number of old governance committee members who would be retained, before opening the other slots to community elections (I, J). In the 6 sites where election of new members took place, diverse groups of stakeholders were present (median: 5.5 stakeholder groups represented, range: 4-7).

Sites A and F did not hold elections because the membership of the governance committee is tied to the Neighborhood Health Committee (see Supplementary file 1) election process and driven by different timing; site H did not have selection criteria, so they postponed elections; and site C decided it was too soon to hold elections.

 In general, the governance committee secretary was responsible for inviting stakeholders to the AGM. In 2 sites (B and I), the only stakeholders invited to the AGM were traditional leaders. Other community stakeholders such as health volunteers, an important cohort of potential candidates for the governance committee, did not know about the meeting. As a result, although some additional stakeholders did find out about the AGM, few people expressed interest in being a candidate for election. The committees at these sites decided to hold another AGM 2 weeks later, at which all key stakeholders were present to elect the new committee.

Table 3 and the next section on committee composition after elections detail how the membership of the committees changed with the elections.

###  Committee Composition After Elections

 Site B elected a completely new set of committee members, while site E re-elected all the old members. Four sites (D, G, I, and J) re-elected some current committee members but replaced others. The average number of committee members remained consistent at 9.3 after re-elections. Average female representation on the committees remained virtually the same (39% at formation, 38% after elections); however, only 2 sites reached 50% female membership, down from 3 at formation. The number of committees with traditional leadership representation also decreased from 7 to 4. All sites retained representation from their associated health centers.

###  Stakeholder Engagement

Table 5 illustrates types of questions asked according to the type of accountability they may have helped to promote.Most of the questions related to financial issues (43%), and accountability for program performance (28%). Twelve percent of the questions were categorized as democratic accountability, that is, questioning how well the representatives were exercising proper authority on behalf of the community. The remainder of the questions (16%) were more informational in nature, where people just wanted to understand how many people were on the committee, or whether someone was being paid. Two questions were about topics not specific to the MWH: for example, one observer asked when the health center would receive a replacement for the clinical officer, and another asked when staff housing construction would be completed. As the committee answered these questions, they were coded under performance accountability.

**Table 5:** Questions From External Stakeholders Asked at AGMs, by Type of Question and Purpose of Accountability

**Type of Accountability**	**Purpose of Accountability**	**Question (Quote)**
**Financial**	Adherence to policies, procedures, and proper reporting	"Do the tailors have a [bank] account, and is it a group or a personal account?"
		"Headmen's contribution has not been mentioned. No matter how little it is, it would be important to know how much was received."
	Cost control, reduce waste, prevent corruption	"Why do you buy stock from district A instead of district B which is nearby and could reduce on costs?"
		"The figures for the workers are not adding up. According to your figures the workers are getting paid less than what you have reported."
	Progress towards stability	"Has the Governance Committee solicited for funding support from elsewhere?"
**Performance**	Adherence to policies and procedures	"Do you have all the trading certificates for your business?"
	Documentation of performance	"Have you evaluated the competency of the tailors after mentorship lessons to determine whether you are making progress or not?"
	Comparison of performance results to targets	"Your report said that the dip tank [for goat rearing] is leaking. What is the way forward?"
	Organizational learning	"The IGA should consider reducing the prices. Market research should be done and check what the prices around are. At the moment your prices are a bit high."
**Democratic**	Satisfaction/responsiveness	"Why have you not implemented the headmen's resolution as was agreed in last quarter's meeting, that maize bran should be given back to customers?"
	Representativeness	"The Agenda states that we are supposed to have elections. Why should we have elections when the current Governance Committee has been trained and they are doing a great job?"
	Transparency	"Why have you prioritized the construction of the verandah at the expense of the toilet, given the cholera situation?"
	Adherence and proper use of authority	"Why do you have same people in both the governance committee and the sub-committees? We all want to be part of the committee!"
**Information**	Understanding procedure	“When did the tuck-shop open?”

Abbreviations: AGMs, annual general meetings; IGA, income-generating activity.

###  Responsiveness of Committee

Table 6 illustrates answers rated as fully, mostly, somewhat, or not responsive to questions. Governance committee members generally had a good understanding of the questions posed by external stakeholders. About 40% of the responses were fully responsive, and an additional 19% were mostly responsive. At times, responses were convoluted or only partially answered the question. Some governance committee members had difficulty responding to questions in sites where they either had not prepared written reports, or where the member presenting the report was not the same person who had prepared the report. This often led to long debates, especially related to finances.

**Table 6:** Examples of Governance Committee Answers to Questions Asked by External Stakeholders at AGMs, by Level of Responsiveness

**Question**	**Answer**	**Observation**
**Fully Responsive**		
You mentioned that you have registered as a cooperative. Who are the members to that cooperative? (Site D)	When registering, we register the GC as the executive for the Cooperative; however, the membership for the cooperative is the community.	Directly and clearly answered the question, providing detail about the role of GC as executive.
Are there responsibilities for the waiting mothers and their companion that we need to know? There are complaints that the mother is being given a lot of work. (Site H)	The MU’s role is to supervise the mothers while they are staying here. It is the responsibility of the waiting mothers and their companions to keep the place clean and ensure that they wash the beddings when they are discharged.	Clearly explained the responsibilities of the mothers and gave information about the role of MU staff. Could have promised to watch for trends/other complaints.
What made other headmen fail to honor their pledges [to support the MWH]? Were they reminded? How many failed to honor their pledges? (Site I)	20 out of 34 headmen honored their pledges. Reminders were sent to those who have not honored their pledges, but to no avail. The Health Centre Committee was mandated to follow-up with the other headmen.	Answered the question, although GC did not know the motivations of the headmen. A follow-up action was identified involving the Health Centre Committee.
**Mostly Responsive**		
What was the cost of building the agro-shop and tuck shop? [Answer: K2300]. Follow-up question: Is that the total amount? (Site G)	No, that only covers blocks and labour cost. The treasurer’ report has more details. Almost all building materials were donated by [the Project].	The question was eventually answered, but the participant needed to ask a follow-up question to understand that the cost was only partial.
Is your job as a GC member voluntary? (Site C)	Yes, it is.	The answer might have explained others who are paid, eg, MU and/or IGA staff, or benefits GC members receive in-kind (training).
Are you making profit or loss at the hammer mill? (Site I)	Yes we are making profit, though we had. breakdown which led us to having more expenditures.	It is not entirely clear if the breakdown explains the size of profit, or if GC thinks hammer mill may not have a profit in the future.
**Somewhat Responsive**		
It appears you recorded a decline in utilization [of the MWH] between December and January 2018. What could be the reason? (Site J)	Farming period could be the cause.	Answer gives a possible reason; however, explanation of the logical connection between farming and use of MWH would be helpful. GC could also mention normal variability in data.
How much money have you incurred in the losses? What is the rationale of proposing to increase the salaries of the workers when you are incurring losses? (Site H)	The workers have been working for a year and so we are proposing, like in any other organization, that there is a yearly increase. The increment is a way of motivating the workers – it is still a plan and it can be shot down.	Did not answer first part of question regarding losses. Did not show an understanding that funds must be available in order to raise salaries.
Your report said that the dip tank is leaking, what is the way forward? (Site C)	At the moment goats are being sprayed.	Answer explains the short-term solution to the problem, but does not explain how the problem will be resolved in future.
**Not Responsive**		
How will the GC as a cooperative be reporting to PACRA? (Site C)	It is important to register with PACRA because we shall be selling goats in bulk.	Did not answer question about the type of reporting required.
Your report is confusing because it is mixing income and expenditure at the same time (Site B)	Yes, I have misplaced the original report. So this was hurriedly done for this meeting.	Did not explain steps that would be taken to find out the answer to the question.
The figures for the workers are not adding up. According to your figures, the workers are getting paid less than what you have reported (Site H)	This could have be a result of miscalculation. Kindly allow us more time to work on a new financial report.	Did not explain how the GC will share the new financial report with the community.

Abbreviations: GC, Governance Committee; IGA, income generating activity; MU, management unit; MWH, maternity waiting home; PACRA, Patents and Companies Registration Agency; AGMs, annual general meetings.

## Discussion

 The aim of this study was to shed light on the structures and processes of community-led governance and accountability for a MWH intervention in rural Zambia. Specifically, we examined open public meetings facilitated by governance committees to see how they promoted “inward transparency,” that is, the ability for community members to see how community leaders were making decisions and managing resources to achieve goals for the public good. We also examined democratic accountability in terms of election of committee members.

 We found that on average, many different stakeholders attended meetings, and reports were transparent. Stakeholders asked probing questions, especially about financial matters, and governance committee members were generally responsive to questions. Half the sites used elections to replace committee members, demonstrating democratic accountability.

These findings are similar to Papp et al, who found that public hearings in Orissa, India, enhanced social accountability by providing participants with a safe space to describe their needs, and helped women and communities to see maternal health services as a right rather than a “kindness.”^25^ The public comment period at open meetings facilitates transparency in 2 ways. It allows officials to respond directly to citizen complaints, but it also raises topics that officials may report back on at a subsequent meeting.^26^ Given the timing of our study, we were unable to measure these less immediate transparency gains. The results of the elections to replace governance committee members in 5 sites also suggest enhanced social accountability. This is similar to findings in Uganda, where communities that participated in a monitoring intervention were more likely to replace governance committee members compared to control sites.^27^

 While AGMs and elections worked generally as expected, the effectiveness of these specific governance activities in promoting inward transparency and accountability was handicapped by committee members’ lack of preparation for the AGM, a narrow focus on financial accountability, and failure of the committees to adopt a strategic performance management perspective.

First, many committees did not perform as expected in preparing for the AGM. Collaboration for community well-being depends on information sharing and mutual goals,^28^ and trust among the different stakeholders can help to harmonize individual interests with community needs.^29^ Yet, several governance committees had incomplete reports and seemed unprepared to answer questions, thus missing opportunities to increase understanding and trust within the community. In addition, lack of preparation meant that elections were not held in 4 of 10 sites, and where elections were held, the proportion of sites with 50% or more women on committees declined, as did representation of traditional leaders. The loss of representation by traditional leaders is significant as the leaders have influence at the village level and can promote use of MWHs.

The second major factor inhibiting transparency and accountability was a narrow focus on financial accountability. External stakeholders asked few questions about non-financial performance, such as the MWH’s ability to deliver quality services, the efficiency, or the committee’s success in promoting organizational growth. The lack of attention to performance transparency includes the failure to incorporate the perspectives of women who had stayed at the MWH. Performance data monitoring and review feature in the accountability framework proposed by the United Nations Commission on Information and Accountability for Women’s and Children’s Health^30^ and can help guide service delivery improvements.^9,31^ Inclusion of the perspectives of key populations — for example, health service users and people living with or affected by disease — is a core governance principle of organizations like the Global Fund,^32^ the World Health Organization (WHO),^33^ and the World Bank.^34^ Participatory approaches in health require power-sharing with health service users in order to be truly transformative.^35,36^

Finally, the lack of strategic management perspective resulted in missed opportunities to promote accountability for responsive actions. For example, AGM participants asked specific questions about the tailoring IGA. While the committee members directly answered these questions, they missed the opportunity to lead the discussion at a higher level: the tailoring IGAs were less successful than other IGAs due to machines that kept breaking down, and there was too little demand for the finished products. The AGM could have been used to reevaluate whether the tailoring IGAs should be continued, given these challenges. In another example, one community member questioned why mothers or their companions had to help with cleaning while staying in the MWH. The committee member explained the current rationale, and the discussion moved on to another topic. Yet, this question points to a need to possibly reconsider the policy, or to sensitize community members about the need for volunteer assistance in managing the MWHs. Similarly, a stakeholder asked about monthly variation in MWH stays. The governance committee member related this to the farming season, implying that women are choosing not to go to the MWH in order to work in the fields. Yet, the governance committee missed the chance to discuss the broader implications for access to the MWH and skilled delivery during the farming season, and strategies to overcome barriers to use the homes during this time. Some of these barriers include having funds to purchase required items for the baby and mother during delivery, and having a relative at home to take care of other children.^22,37-39^

 Despite project-sponsored governance and financial trainings, as well as mentorship over a period of 24 months, more effort, or different approaches, were needed to increase the members’ capacity for community governance and accountability. Several factors could improve the open meeting and election process in the future and in other settings, including better meeting preparation and facilitation, strengthening performance data, and focused mentoring. These factors should be considered by policy-makers, program managers, and development partners interested in promoting community-owned MWHs. The factors may even help support the health services locally beyond the MWHs.

###  Preparing for Meetings and Meeting Facilitation

The project provided general guidelines for the AGM and preparatory meetings, but the committees required further normative guidance. Based on this experience, the project created written templates for the AGM Agenda, Activity, and Finance Reports (see Supplementary file 3), and a checklist of stakeholders to invite to the meeting. Written reporting templates can help ensure that key information is included. Program managers should assure that committee members understand the reports. A run-through of the meeting, using a set of possible questions derived from previous meetings, may be helpful. The members need to be prepared to explain their role, give more details behind decisions, and describe their vision for the future. Committee members also need to develop skills in how to anticipate questions and manage discussion among community members attending the public meetings. Holding a “mock” meeting as part of preparatory training, with project staff asking potentially contentious questions, could help committee members gain confidence, apply judgement, and develop meeting management skills.^40,41^

###  Strengthening Performance Data

It may be helpful to ask interested stakeholders to evaluate governance committee and MWH performance prior to the open public meeting and elections. This would better prepare meeting participants to discuss results and vote. Indicators for the governance committee could include governance committee members’ attendance at quarterly meetings, and existence of good meeting minutes. MWH performance metrics might include average length of stay, occupancy rate, and satisfaction ratings from women using it. This could help build a culture of organizational learning focused on opportunities for growth and change.^42^

###  Focused Mentoring

 A barrier to democratic accountability in some sites was a lack of community understanding of the purpose of elections. In 2 communities, stakeholders wondered why elections were needed if the current governance committee was performing well. Methodologies for electing governance committee members were complicated by the difficulty of communication across the multiple villages served by each site. Technical assistance and mentoring provided by government or development partners may help contextually adapt the election models and assure deeper understanding of the governance committee’s roles and responsibilities. This is especially important as members are replaced through elections. Our analysis suggests that this technical assistance may be needed for a longer period than 24 months, with implications for program costs. A critical question is whether the extensive inputs in the committees are likely to be sustained if the donor funds are not available. Models for local technical assistance could be explored to extend mentoring for community leaders; for example, the Ministry of Health could partner with the Ministry of Community Development and Social Welfare and the Ministry of Gender. It is within the scope of influence of these government agencies to support and empower community and women leaders.

The AGMs did not meet expectations related to representation of the views of women of reproductive age. None of the sites designed election processes that explicitly encouraged affirmative action with regard to gender representation. This is something to address in the future, perhaps through different models such as strengthening the role of health facility staff in MWH governance^43^ or incorporating user feedback interviews into AGM planning.^44^ Researchers in Benin found that conducting personalized feedback interviews with women and their companions after receiving maternal care, helped women to overcome institutionalized norms of passivity and to redress injustices they experienced.^36^ The data thus obtained can be used to improve maternal care. Increasing the voice of users could increase democratic accountability by encouraging critical reflection on the part of the governance committee and providing motivation for change.^36^

Additional research could explore other models of accountability in the context of community-governed MWHs. For example, McDonald argues that the qualities of commitment, cultural humility, and partnership are central to social accountability.^45^ These qualities are demonstrated when governance committee members are embedded in the community, are respectful of the mores of the local culture, and exhibit an egalitarian approach to solving problems together with the community. Longer-term research could try to measure how the governance committees exhibit these qualities over time, and how to strengthen them to further support social accountability.

Our study did not specifically examine the role of traditional leaders vis-à-vis other formal leaders in the establishment of the governance committees and in the oversight process. This would be an important area of exploration in future research. It is possible that involving traditional leaders more intentionally from the start could have strengthened the performance of the committees, though it is also possible that traditional leaders could exert coercive pressures on MWH governance decisions. Qualitative data from Zambia suggests that traditional leaders are imposing financial penalties on women who deliver at home.^46^ This was the case in Malawi, where traditional leaders supported maternal, newborn, and child health goals through a utilitarian, top-down model that included fear, coercion, and punishment as tools for policy implementation.^47^

###  Limitations

This study had several limitations. In observing meetings, we were unable to record the gender, occupation, or other demographic information for citizens raising questions at meetings, so we cannot evaluate possible power imbalances that may have limited certain perspectives from being represented, a problem suggested in other studies.^4^ In addition, using project staff as meeting observers may have introduced bias, as these staff had been mentoring the committees for several years. Their familiarity gave them background knowledge, but could have caused them to emphasize certain aspects of meetings or overlook others, based on the site. This limitation was somewhat mitigated by using checklists to evaluate transparency of documents, and by requiring staff to write down every question asked and answered. The study may also have been biased by the Hawthorne effect^48^: committee members may have changed their behavior in response to the interest or attention of the project staff who observed the annual meetings. Finally, the analysis is based primarily on project record review and the quality of the reports varied.

## Conclusion

Our paper provides insights on the contextual conditions for implementation of community-level accountability interventions to increase internal transparency, something that has been lacking in other studies.^9^ The findings suggest that open public meetings and elections can be an effective mechanism to increase accountability for health institutions at the community level; however, governance committee members need support and mentorship to adequately prepare for and facilitate meetings and elections. The process of educating committee members and stakeholders to participate in community-led governance structures takes time and should not be seen as a one-time investment. Detailed study of open meetings can be helpful to reveal gaps in understanding of policies and performance, and the data collected can be used to develop contextually adapted training and leadership development programs going forward.

## Acknowledgements

 The authors would like to thank and acknowledge the work of the Maternity Homes Alliance, the Ministry of Health, the District and Provincial Health offices, and the Chiefs of the study areas. Special thanks to Kaluba Mataka for her guidance, and Karen Hussmann for her comments on an earlier version of this manuscript.

## Ethical issues

 Ethical approval was obtained from the Institutional Review Board at Boston University (Ref No. H-35321) and from the ERES Converge IRB in Zambia (Ref. No. 2016-June023). Permission was granted by the Zambia Ministry of Health to conduct research at study sites. Informed consent was not required for this particular study since only project records were used for this analysis.

## Competing interests

 All authors report institutional grants from MSD for Mothers, the Bill & Melinda Gates Foundation and The ELMA Foundation during the conduct of the study.

## Authors’ contributions

 NAS is the principal investigator and TV is a co-investigator of the overall MWHs evaluation. NAS led the design and implementation of the intervention and TV provided guidance for the governance aspect. MB and VIRS provided supervision and mentorship to the governance committee members and kept project records of the activities. TN supervised all field work. TV, RMF, and JLK coded, analyzed, and interpreted data from the project records with assistance from MB. TV and RMF primarily drafted and revised early version of the manuscript, and JLK provided substantial revisions to the later drafts. All authors provided critical revisions to the manuscript.

## Disclaimer

The funders had no role in study design, data collection and analysis, decision to publish, or preparation of the manuscript. The content is solely the responsibility of the authors and does not reflect positions or policies of *MSD for Mothers*, the Bill & Melinda Gates Foundation, or The ELMA Foundation.

## Funding

This program was developed and implemented in collaboration with *MSD for Mothers*, MSD’s 10-year, $500 million initiative to help create a world where no woman dies giving life. *MSD for Mothers *is an initiative of Merck & Co., Inc., Kenilworth, NJ, USA [grant number: MRK 1846-06500.COL]. The development of this article was additionally supported in part by the Bill & Melinda Gates Foundation [grant number: OPP1130329] and The ELMA Foundation [grant number: ELMA-15-F0017].

## Authors’ affiliations

^1^School of Nursing and Health Professions, University of San Francisco, San Francisco, CA, USA. ^2^Department of Global Health, Boston University School of Public Health, Boston, MA, USA. ^3^Department of Research, Right to Care Zambia, Lusaka, Zambia. ^4^Department of Programs, Amref Health Africa, Lusaka, Zambia.

## Supplementary files


Supplementary file 1. Description of Stakeholder Groups Attending Annual General Meeting, Zambia.

Supplementary file 2. Reporting Transparency Criteria.

Supplementary file 3. Templates for the Annual General Meeting Agenda, Activity Report, and Finance Report.
