# Supplementary material for: Using Open Public Meetings and Elections to Promote Inward Transparency and Accountability: Lessons From Zambia
Source: Int J Health Policy Manag. 2020 Jun 23;11(2):160–72. doi: 10.34172/ijhpm.2020.84 (PMC9278615; doi:10.34172/ijhpm.2020.84)
Supplement: Supplementary file 1 — Description of Stakeholder Groups Attending Annual General Meeting, Zambia. [file ijhpm-11-160-s001.pdf]

Supplementary file 1. Description of Stakeholder Groups Attending Annual General Meeting, Zambia

(1) Traditional leadership

Traditional leadership consists of chief representatives, senior headmen and headmen/headwomen. They are:

- Custodians of natural resources such as traditional land
- Facilitators of social development for their people
- Responsible for settling disputes in their communities
- Guardianship of traditional heritage such as norms, culture, beliefs and values
- Linkage between government and the people

(2) Health facility staff

Health facility staff are skilled health professionals employed by the central government at rural health centers, in this context. They are trained as midwives, nurses, clinical officers, and environmental health technicians. They provide services in primary healthcare, nutrition, maternal and child health, clinical screening, and referrals.

(3) Health volunteers

Health facility-affiliated volunteers play an integral role in primary healthcare through multiple cadres, including community health workers, Neighborhood Health Committees, Safe Motherhood Action Groups, Traditional Birth Attendants, counselors, malaria agents etc. They provide:

- Community Health education and outreach services
- Basic HIV/AIDS counselling and support services
- Basic patient screening at the facility and health posts
- Data collection and relay of health-related information to policy-makers
- Connections between the formal health system and the people
- Community mobilization

(4) Church

The church is a key stakeholder in the community. Its role includes:

- Meeting the spiritual, emotional and physical needs of people
- Providing education and health services (2 health facilities in our study are mission owned)
- Advocating for social and economic justice
- Partnering in development (eg, by building community structures, such as a cooking facility at one MWH)

(5) School

Schools represent a social institution responsible for broad-based development of pupils. Schools in the rural Zambian context are intricately involved in the health of their communities:

- Schools are used to relay information between health centers and parents through pupils.
- School teachers participate in community health development by sitting on local health committees responsible for implementing and overseeing programs
- Schools teach good health practices and hygiene to their pupils

(6) Government

Secular government representatives at the local level include civil councilors.

(7) Others

- Stakeholders categorized as "others'' include observers, youth, farmers, and men and women who never introduced themselves as falling in any category listed above.
