# Supplementary material for: Using Open Public Meetings and Elections to Promote Inward Transparency and Accountability: Lessons From Zambia
Source: Int J Health Policy Manag. 2020 Jun 23;11(2):160–72. doi: 10.34172/ijhpm.2020.84 (PMC9278615; doi:10.34172/ijhpm.2020.84)
Supplement: Supplementary file 2 — Reporting Transparency Criteria. [file ijhpm-11-160-s002.pdf]

Supplementary file 2. Reporting Transparency Criteria

1) Annual General Meeting Agenda

A written agenda should include time for the following elements:

1) Opening remarks and prayer
2) Introductions
3) Activity (Chairman's) report presentation
4) Feedback/discussion/vote to accept activity report
5) Finance (Treasurer's) report presentation
6) Feedback/discussion/vote to approve finance report
7) Sub-committee reports and discussion
8) Headmen's report or report from other formal stakeholders or observers
9) Election criteria presentation/discussion
10) Retention or election of new governance committee members

2) Activity Report

An activity report should meet the following criteria for transparency:

1) Written report (not only verbal)
2) Reporting period is clearly specified
3) Leadership executive summary is provided
4) Planned activities for the reporting period are described (what the governance committee planned to accomplish)
5) Implemented activities are described (the activities that were actually implemented, and results of those activities)

6) Unimplemented activities are described (what planned activities were not implemented, and why)

7) Main achievements during the reporting period are summarized

8) Challenges are discussed

9) Includes recommendations to surmount challenges

10) Includes a conclusion and plans for going forward

3) Finance Report

A financial report should meet the following criteria for transparency:

1) Written report (not only verbal)
2) Reporting period specified
3) Report includes all streams of income and amounts received during period
4) Report includes all categories of expenditure and amounts spent during period
5) Report includes profit or loss analysis
