# Supplementary material for: Using Open Public Meetings and Elections to Promote Inward Transparency and Accountability: Lessons From Zambia
Source: Int J Health Policy Manag. 2020 Jun 23;11(2):160–72. doi: 10.34172/ijhpm.2020.84 (PMC9278615; doi:10.34172/ijhpm.2020.84)
Supplement: Supplementary file 3 — Templates for the Annual General Meeting Agenda, Activity Report, and Finance Report. [file ijhpm-11-160-s003.pdf]

Supplementary file 3. Templates for the Annual General Meeting Agenda, Activity Report, and Finance Report

(1) AGM Agenda Template:

ANNUAL GENERAL MEETING AGENDA
Name of Committee/Association

Time, Date & Venue

1. Opening of Meeting
2. Apologies
3. Confirmation of Minutes of previous Annual General Meeting
4. Presentation of Annual Report (chairperson's report)
2. Adoption of Annual Report
3. Presentation of Treasurer's report
4. Adoption of treasurer's report
5. Tenure/Election of New Executive/committee
6. Vote of thanks to outgoing Executive
7. Determination of Annual Membership Fee (optional)
8. Notice/s of Motion
9. Urgent general business
10. Closure

(2) Activity Report Template:

ACTIVITY REPORT TEMPLATE

NAME: ……………………………………………..……. DISTRICT: …………………………………………………………….

a) Leadership

NAME	SEX	DATE JOINED	POSITION	SUB-COMMITTEE

b) Planned activities

c) Implemented activities

d) Progress on specific MWH activities

1. Maternity waiting home status, assets and activities

Status:

……

Assets:

……

Activities:

……….

Utilization rates:

………

2. IGA

Type of IGA:

………

……..…..

Date started:

………

…………

Achievements:

………

………

Challenges:

………

……………

Recommendations:

……………………………………………………………..……………………………………………………………

3. Keyhole gardening:

How many have been constructed?

………………………………………………………………………………………………..

How many are fenced? ………………...

How many are currently planted?

…………………………………………………………………………………………………

Number of harvests since construction?

……………………………………………………………………………………….

Challenges:

……

……………

Recommendations:

……

Key achievements during the period under review:

……
……………………………………

Key challenges during the period under review:

……
……………………………………

(3) Finance Report Template:

Reporting period (Month - Month Year)

A	**Income (From hammer mill, maize bran & Maize sales)**		
	**Community contribution**	-	
**Total**			
B	**Expenditure**		**Comments**
1	Diesel		
2	Servicing		
3	Wire fence and poles		
4	Construction labor		
5	Stipends		
6	Purchase of blocks		
7	Cooperative registration		
8	Mother's shelter support		
9	Transport		
10	Commemorations of world aids day		
11	Food-meetings		
12	Exchange visit		
13	Empty bags and grease		
14	Emergency fund		
15	Depreciation		
16	Mother's shelter Assets savings		
C	**Total expenditure**	-	
D	**Variance**	-	
E	**Cash at bank**		

Prepared by:…………………………………………..

Date:……………………………..

Reviewed by:…………………………………………..

Date:………………………………
